# Novel heterozygous pathogenic variants in *CHUK* in a patient with AEC-like phenotype, immune deficiencies and 1q21.1 microdeletion syndrome: a case report

**DOI:** 10.1186/s12881-018-0556-2

**Published:** 2018-03-09

**Authors:** Maxime Cadieux-Dion, Nicole P. Safina, Kendra Engleman, Carol Saunders, Elena Repnikova, Nikita Raje, Kristi Canty, Emily Farrow, Neil Miller, Lee Zellmer, Isabelle Thiffault

**Affiliations:** 1 Center for Pediatric Genomic Medicine, Children’s Mercy Hospital, Kansas City, MO USA; 2 Division of Clinical Genetics, Children’s Mercy Hospital, Kansas City, MO USA; 3 Department of Pathology and Laboratory Medicine, Children’s Mercy Hospitals, Kansas City, MO USA; 4 Pediatric Allergy, Asthma and Immunology Clinic, Children’s Mercy Hospitals, Kansas City, MO USA; 5 Dermatology Clinic, Children’s Mercy Hospitals, Kansas City, MO USA; 6 Department of Pediatrics, Children’s Mercy Hospital, Kansas City, MO USA; 7 University of Missouri Kansas City, School of Medicine, Kansas City, MO USA

**Keywords:** AEC, Bartsocas–Papas syndrome, CHUK, Cocoon syndrome, 1q21.1 microdeletion syndrome

## Abstract

**Background:**

Ectodermal dysplasias (ED) are a group of diseases that affects the development or function of the teeth, hair, nails and exocrine and sebaceous glands. One type of ED, ankyloblepharon-ectodermal defects-cleft lip/palate syndrome (AEC or Hay-Wells syndrome), is an autosomal dominant disease characterized by the presence of skin erosions affecting the palms, soles and scalp. Other clinical manifestations include ankyloblepharon filiforme adnatum, cleft lip, cleft palate, craniofacial abnormalities and ectodermal defects such as sparse wiry hair, nail changes, dental changes, and subjective hypohydrosis.

**Case presentation:**

We describe a patient presenting clinical features reminiscent of AEC syndrome in addition to recurrent infections suggestive of immune deficiency. Genetic testing for *TP63*, *IRF6* and *RIPK4* was negative. Microarray analysis revealed a 2 MB deletion on chromosome 1 (1q21.1q21.2). Clinical exome sequencing uncovered compound heterozygous variants in *CHUK*; a maternally-inherited frameshift variant (c.1365del, p.Arg457Aspfs*6) and a *de novo* missense variant (c.1388C > A, p.Thr463Lys) on the paternal allele.

**Conclusions:**

To our knowledge, this is the fourth family reported with *CHUK*-deficiency and the second patient with immune abnormalities. This is the first case of *CHUK*-deficiency with compound heterozygous pathogenic variants, including one variant that arose *de novo*. In comparison to cases found in the literature, this patient demonstrates a less severe phenotype than previously described.

**Electronic supplementary material:**

The online version of this article (10.1186/s12881-018-0556-2) contains supplementary material, which is available to authorized users.

## Background

Ectodermal dysplasias (ED, OMIM:604292) are a group of diseases affecting the teeth, hair, nails and exocrine and sebaceous glands. In some cases, part of the skin, eyes, inner ears, fingers, toes and central nervous system can also be affected. There are approximately 150 different types of ED, the most commonly recognized syndromes being the ectrodactyly-ectodermal dysplasia-clefting syndrome (EEC, OMIM: 129900), Rapp-Hodgkin syndrome (OMIM: 129400) and ankyloblepharon-ectodermal defects-cleft lip/palate syndrome (AEC, OMIM:106260) [1]. AEC syndrome, also known as Hay-Wells syndrome, is caused by heterozygous pathogenic variants in *TP63* [2, 3]*.* A classical feature of AEC syndrome is the presence of skin erosions affecting the palms, soles and scalp. Other clinical manifestations include ankyloblepharon filiforme adnatum, cleft lip, cleft palate, craniofacial abnormalities, and ectodermal defects such as sparse wiry hair, nail changes, dental changes, and subjective hypohydrosis [4–6].

*IRF6*-related disorders are a group of inherited disorders associated with heterozygous pathogenic variants in *IRF6,* including Van der Woude syndrome (VWS, OMIM:119300) and popliteal pterygium syndrome (PPS, OMIM:119500). VWS is characterized by orofacial clefting and lip pits whereas PPS is characterized by similar lip/palate abnormalities in combination with ankyloblepharon in some cases, as well as digital and genital abnormalities [7, 8]. Bartsocas-Papas syndrome (BPS, OMIM: 263650), a severe form of PPS, is associated with homozygous pathogenic variants in *RIPK4* and *CHUK* [9–11]. Recently, a *de novo* missense variant in *CHUK* was reported in one patient with ectodermal dysplasia, orofacial clefting, limb anomalies and hypogammaglobulinemia [12].

Copy number variants affecting the 1q21.1 region have been associated with genomic disorders. Phenotypic features of 1q21.1 deletion syndrome include microcephaly (50%), mild intellectual disability (30%), mildly dysmorphic facial features, and eye abnormalities (26%). Other findings can include cardiac defects, genitourinary anomalies, skeletal malformations, and seizures (~ 15%). Psychiatric and behavioral abnormalities can include autism spectrum disorders, attention deficit hyperactivity disorder, and sleep disturbances (OMIM: 612474). The majority of microdeletions are inherited, and incomplete penetrance and variable expressivity have been noted [13–15]. In this report, we describe, for the first time, a patient with compound heterozygous variants in *CHUK*. Interestingly, one variant arose *de novo*. To our knowledge, this is the second patient with *CHUK*-deficiency and immune abnormalities associated with *de novo* variant in *CHUK*. However, based on our data, it is unclear if, in some cases, *de novo* heterozygous *CHUK* variants are sufficient to cause disease. Clinical features of the patient are consistent, although less severe, with previously reported cases. This patient is also carrier of a 2 MB deletion on chromosome 1 which might contribute to some of his features.

## Case presentation

**Fig. 1:**
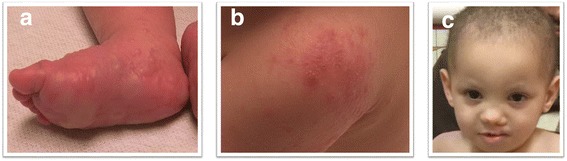
Clinical photographs of the index patient. **a**-**b** Mild 2^nd^, 3^rd^ toe syndactyly, eczema, recurrent onychomadesis, recurrent skin infections causing desquamation; **c** the patient at 2 years of age - sparse hair, eyelashes and eyebrows, depressed flat nasal bridge, hypoplastic alae nasi, thin vermillion border, mild epicanthus, ankyloblepharon and unilateral left cleft lip and palate s/p repair

Our patient is a male born to healthy non consanguineous parents weighing 2.375 kg, measuring 48 inches at birth. Maternal and paternal age were 27 and 25 years old, respectively. During the pregnancy there were no exposures to drugs, alcohol, tobacco or medications. The fetal movements were described as normal up until approximately 32 weeks gestation, when they were noted to be decreased. He was delivered by induced vaginal delivery at 37 + 4 weeks gestation due to intrauterine growth retardation and reduced fetal movements. He was transferred to the Children’s Mercy Hospital Neonatal Intensive Care Unit (NICU) on day 1 of life due to cleft lip and palate and ankyloblepharon filiforme adnatum. Physical examination revealed sparse eyelashes and eyebrows, hypoplasia of the teeth, abnormal palmar creases, 5^th^ finger clinodactyly, mild 2^nd^, 3^rd^ toe syndactyly and hypohidrosis (Fig. 1). The patient had recurrent bacterial and viral infections. His infections included recurrent otitis media despite bilateral myringotomy and tube placement, *Staphylococcus aureus* impetigo, coxsackie hand foot mouth disease, recurrent upper and lower respiratory infections including respiratory syncytial virus (RSV) bronchiolitis and multiple episodes of non-RSV viral bronchiolitis. His immune work up showed mild abnormalities including low immunoglobulin (Ig) M (31 mg/dL) and low normal IgG levels (355 mg/dL). His IgA was normal (17 mg/dL). His lymphocyte subsets showed normal T cells (CD3; 1860 mm^3^) but mildly low CD4 (1333 mm^3^) and CD8 (372 mm^3^) subsets. The patient’s developmental history was appropriate. He had a head ultrasound, abdominal ultrasound, echocardiogram and bone survey which were unremarkable. This clinical presentation led to the suspicion of an ectodermal dysplasia syndrome such as AEC syndrome, Bartsocas-Papas syndrome or Van der Woude syndrome. Gene testing for *TP63*, *RIPK4* and *IRF6* was negative. Microarray analysis revealed a 2 MB deletion on chromosome 1 encompassing 18 genes (arr [hg19] 1q21.1q21.2 (145,885,645–147,929,115)). Parental studies were requested but not performed.

**Fig. 2:**
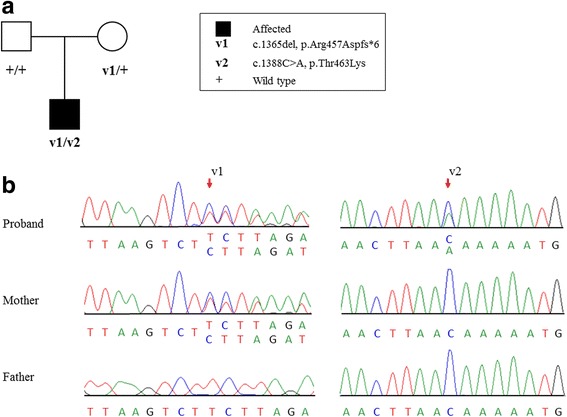
*CHUK* pathogenic variants and segregation studies. **a** Family pedigree showing segregation studies; **b** Sanger sequencing indicates that the c.1365del variant was maternally-inherited. The c.1388C > A (p.Thr463Lys) was not detected in either parental sample. Paternity was confirmed, indicating that the c.1388C > A variant arose *de novo*

Clinical exome sequencing was performed on the affected individual with methods as previously published [16–18]. Variants were filtered to 1% minor allele frequency, then prioritized by the American College of Medical Genetics (ACMG) categorization [19], OMIM identity and phenotypic assessment. This individual was found to be compound heterozygous for a frameshift variant c.1365del (p.Arg457Aspfs*6) and a missense variant c.1388C > A (p.Thr463Lys) in *CHUK* (NM_001278.3) (Fig. 2a). Both variants are located in exon 13 and occurred *in trans*, as visualized by the Integrative Genomics Viewer (IGV) tool (Additional file 1: Figure S1) [20, 21]. Sanger sequencing confirmed that the p.Arg457Aspfs*6 variant was maternally-inherited and the p.Thr463Lys was not detected in either parental sample (Fig. 2b). Paternity was confirmed using short-tandem repeat analysis. This indicates that the p.Thr463Lys variant arose *de novo*, but germline mosaicism in the father can’t be excluded. These variants were absent from population databases.

## Discussion & conclusion

Pathogenic variants in *CHUK* have been reported in 3 families to date (Table 1; Additional file 2: Figure S2): In 2010, Lahtela et al., described a Finnish family in which a homozygous loss of function variant in *CHUK* (c.1264C > T; p.Gln422*) was associated with Cocoon syndrome, an autosomal recessive lethal condition characterized by severe fetal malformations. Prenatal ultrasound of 2 fetuses revealed an abnormal cyst in the cranial region, a large defect in the craniofacial area, an omphalocele and immotile and hypoplastic limbs. Abnormalities of the heart, lungs, skin, bones and skeletal muscles were also observed. Both parents were heterozygous for this variant and genealogical analysis revealed a common ancestor [22]. In 2015, Leslie et al., reported a homozygous variant in the splice acceptor site of exon 10 (c.934-2A > G) in a female patient with Bartsocas-Papas syndrome born to healthy first degree cousins. Clinical manifestations included alopecia totalis (with absent eyebrows and eyelashes), wide cranial suture and fontanelle, nose and ear dysmorphisms, bilateral microophthalmia, ankyloblepharon, bilateral cleft lip and palate, genital hypoplasia, popliteal webs and skeletal abnormalities [11]. In 2017, Khandelwal et al., reported a 10-year-old female born to non-consanguineous Caucasian parents with a *de novo* missense variant in *CHUK* (c.425A > G, p.His142Arg). Clinical features of the patient included sparse hair, absent eyebrows and eyelashes, ankyloblepharon and dysplastic nails. X-rays of the hands and feet showed complex anomalies consisting of, among others, hypoplastic thumbs and 3^rd^–5^th^ toe syndactyly. Other features included posterior cleft palate, retrognathia, buccal synechia, hypoplastic external genitalia, conical and fragile primary teeth and short stature (height -3.5SD and weight -3SD). Her development was marked by growth retardation, gastrointestinal reflux with swallowing problems and lower respiratory tract infections. She also had hypogammaglobulinemia. To our knowledge, a second pathogenic variant was not detected in this patient, but additional screening methods such as deletion/duplication analysis were not performed [12].

**Table 1:** Comparison of clinical features of patients with variants in *CHUK* reported in the literature and in this report

	Lahtela et al., 2010 [22]	Leslie et al., 2015 [11]	Khandelwal et al., 2017 [12]	This study
Mode of inheritance	AR	AR	AD (?)	AR (with *de novo* paternal variant)
Variant(s)	hom c.1264C > T (p.Gln422*)	hom c.934-2A > G (p.?)	het *de novo* c.425A > G (p.His142Arg)	comp het c.1388C > A (p.Thr463Lys) / c.1365del (p.Arg457Aspfs*6)
Age	14 and 13 weeks gestation	Infant	10 years	30 months
Sex	Females (2 fetuses)	Female	Female	Male
Family history	Negative	Negative	Negative	Negative
Hair	n.a	Alopecia totalis, absent eyebrows and eyelashes	Sparse hair, absent eyebrows and eyelashes	Sparse short scalp hair, sparse eyebrows
Cranium	Underdeveloped skull bones, abnormal cyst	Wide cranial suture and anterior fontanelle, prominent occiput	n.a	Normal
Ears	n.a	Low set with overfolded helices	n.a	Normal
Eye	Missing eyes, hypoplastic eyeballs	Bilateral microphtalmia, ankyloblepharon, cloudy corneas	Ankyloblepharon	Mild epicanthus, ankyloblepharon
Mouth	Abnormal orifice covered with skin	Cleft lip/palate (bilateral), intraoral bands	Cleft palate (posterior), buccal synechia	Cleft lip/palate (unilateral left)
Nose	Sharp protrusion	Distorted, absent alae nasi	Hypoplastic alae nasi	Depressed flat nasal bridge, alae nasi hypoplasia
Chin	n.a	Micrognatia	Retrognathia	n.a
Chest	n.a	Hypoplastic nipples, short sternum	n.a	Normal
Abdomen	Omphalocele	High umbilical stump, umbilical cord fused to the abdominal wall. No organomegaly	n.a	Soft without organomegaly
Upper extremities	Hypoplastic, encased under skin	Short, bilateral cubital webs	n.a	Normal appearance
Hands	n.a	Small, bilateral syndactyly	Abnormal	Significant 5^th^ finger clinodactyly. Single creases bilaterally
Lower extremities	Hypoplastic, encased under skin	Very short with popliteal webs extending from the upper thigh to the feet	n.a	Normal appearance. No pterygium
Feet	n.a	Fused forefeet	Bilateral toes syndactyly of three rays with dysmorphic phalanges	Mild 2^nd^, 3^rd^ toe syndactyly
Skin/tegument	Abnormal transparent skin	Skin tags (scalp, right eyelid, umbilical cord, vagina)	Dysplastic nails	Light skin coloration. Mild eczema
Genitalia	n.a	Hypoplasia of the labia majora, labia minora, and clitoris	Hypoplastic external genitalia	n.a
Skeletal survey	Hypoplastic bones	Three metacarpal bones, hypoplasia of proximal phalanges, aplasia of distal phalanges (bilateral), absent foot bones (except the talus; left side), absent calcaneus, absent tarsal bones, hypoplasia of the foot phalanges (right side)	Hypoplastic thumbs and first metacarpals, four metatarsal bones with large proximal extremity of the 4^th^ ray (left side) and fusion between the 4^th^ and the 5^th^ rays (right side)	n.a

Legend: *n.a* information not available, *E* Embryonic

*Describe a stop codon. It is part of the nomenclature convention

The *CHUK* gene encodes for Ikka (Inhibitor of nuclear factor kappa-B kinase subunit alpha), a catalytic subunit of the multiprotein complex IbK kinase. Studies in mice show that the Ikka protein is ubiquitously expressed with the highest levels in the developing spine, limb buds and head. It plays an important role in limb development, apoptosis of interdigital tissue and proliferation and differentiation of epidermal keratinocytes. Embryos from the Ikka-deficient mice developed to term, but died shortly after birth. The fetuses displayed several skeletal abnormalities affecting the size and the morphology of the spine, skull, forepaws and the hindpaws. Limb bones were relatively smaller and of normal shape. Microscopic evaluation of the skin revealed hyperplasia of the suprabasal layer (stratum spinosum) and absent stratum granulosum and stratum corneum. Mice heterozygous for the *CHUK* gene deletion are normal, viable and fertile [23].

In this report, we describe a male patient presenting with an AEC syndrome-like phenotype and recurrent infections suggestive of immune deficiency. Targeted sequencing of *TP63*, *RIPK4* and *IRF6* was negative. Microarray analysis identified a 2 MB deletion on chromosome 1 covering the distal part of the 1q21.1 region deletion. Although this pathogenic deletion is unlikely to account for all the clinical features of the patient, it could contribute to his dysmorphic facial features, small size and failure to thrive (Fig. 1, Additional file 3: Figure S3). Additionally, exome sequencing revealed that he was compound heterozygous for two novel variants in *CHUK*. The c.1365del (p.Arg457Aspfs*6) frameshift variant, was inherited from his unaffected mother and the c.1388C > A (p.Thr463Lys) missense variant arose *de novo*. This genotype is compatible with autosomal recessive inheritance and consistent with previously reported families [11, 22]. To our knowledge, only one patient has been reported so far with a *de novo* missense variant in *CHUK* [12]. Since deletion/duplication testing was not performed, the presence of a second undetected variant cannot be ruled out. Interestingly, our patient shares several clinical features with this individual. However, skeletal defects appeared less severe in our patient and we cannot rule out progressive hypogammaglobulinemia needing Ig replacement at follow up. Therefore, based on our findings, it is unclear if, in some cases, the inheritance pattern could be dominant and that *de novo* heterozygous *CHUK* variants are sufficient to cause the disease. Even if the majority of pathogenic *de novo* variants are involved in dominant genetic disorders, there are growing examples of recessive disorders that can be caused by the combination of an inherited variant on one allele and a *de novo* variant on the other.

## Additional files

Additional file 1: **Figure S1.** DNA alignment of NGS data using Integrative Genomics Viewer (IGV). IGV snapshot of exon 13 of *CHUK* gene (NM_001278.3), located on chromosome 10, showing that the two variants (c.1365del, p.Arg457Aspfs*6; c.1388C > A, p.Thr463Lys) are present on different reads, indicating that they occurred on different chromosome (*in trans*). (TIFF 307 kb)

Additional file 2: **Figure S2.** The protein is composed of a protein kinase domain (orange), a leucine zipper region (green) and the NEMO binding-region (blue). Missense (gray) and loss of function (red) pathogenic variants found in the *CHUK* gene. Reported in this study (Bold). (TIFF 128 kb)

Additional file 3: **Figure S3.** Growth chart. A) Weight for age (kg). B) Length for age (cm). (TIFF 602 kb)

## Abbreviations

ACMG: American College of Medical Genetics
AEC: Ankyloblepharon-ectodermal defects-cleft lip/palate syndrome
BPS: Bartsocas-Papas syndrome
ED: Ectodermal dysplasia
EEC: Ectrodactyly-ectodermal dysplasia-clefting syndrome
Ig: Immunoglobulin
IGV: Integrative Genomics Viewer
NICU: Neonatal Intensive Care Unit
OMIM: Online Mendelian Inheritance in Man
PPS: Popliteal pterygium syndrome
RSV: Respiratory syncytial virus
VWS: Van der Woude syndrome
