# Supplementary figures and images for: Novel heterozygous pathogenic variants in CHUK in a patient with AEC-like phenotype, immune deficiencies and 1q21.1 microdeletion syndrome: a case report
Source: BMC Med Genet. 2018 Mar 9;19:41. doi: 10.1186/s12881-018-0556-2 (PMC5845372; doi:10.1186/s12881-018-0556-2)

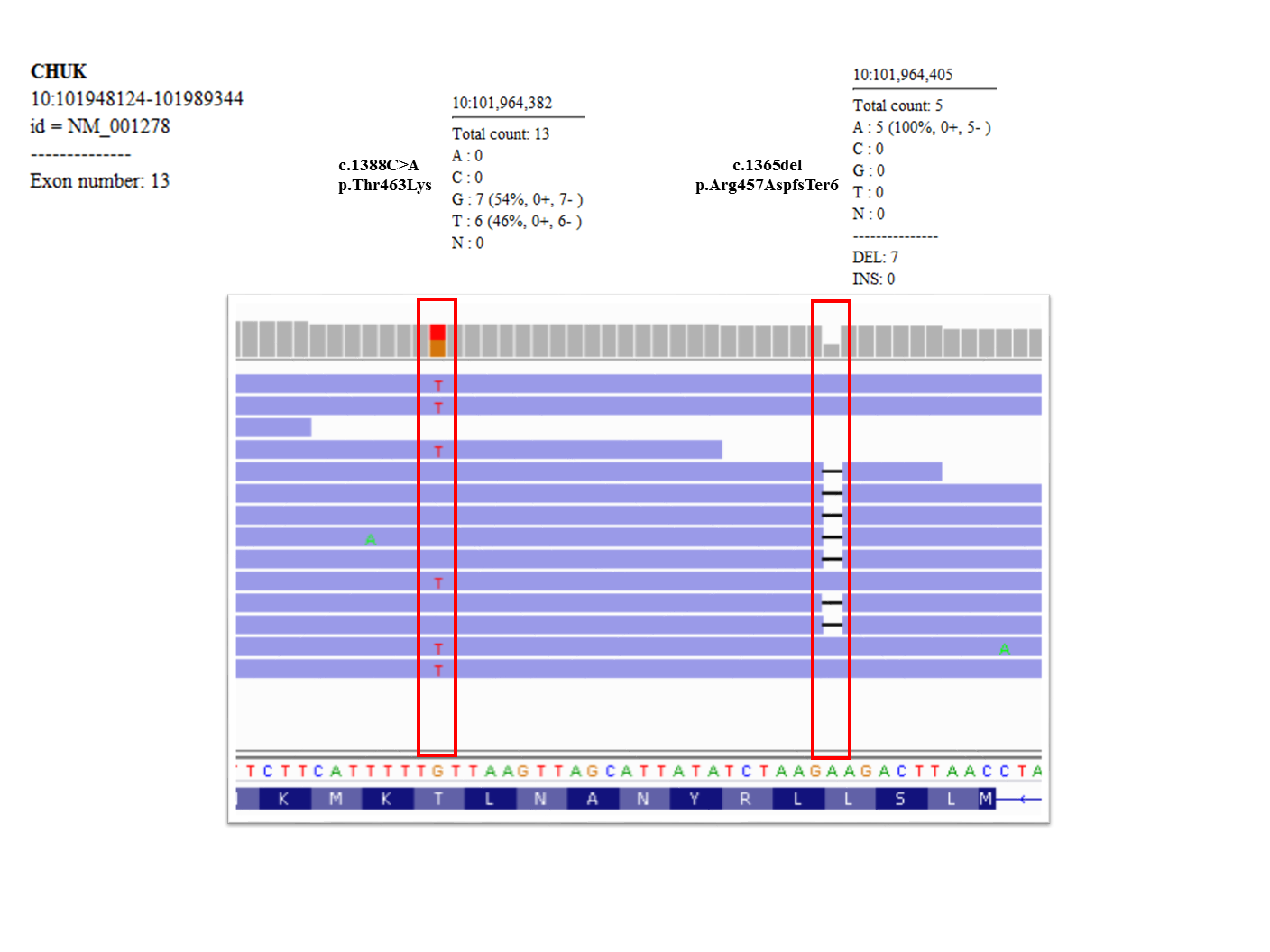

Supplement: Supplementary file 1 — Figure S1. DNA alignment of NGS data using Integrative Genomics Viewer (IGV). IGV snapshot of exon 13 of CHUK gene (NM_001278.3), located on chromosome 10, showing that the two variants (c.1365del, p.Arg457Aspfs*6; c.1388C > A, p.Thr463Lys) are present on different reads, indicating that they occurred on different chromosome (in trans). (TIFF 307 kb) [file 12881_2018_556_MOESM1_ESM.tif]

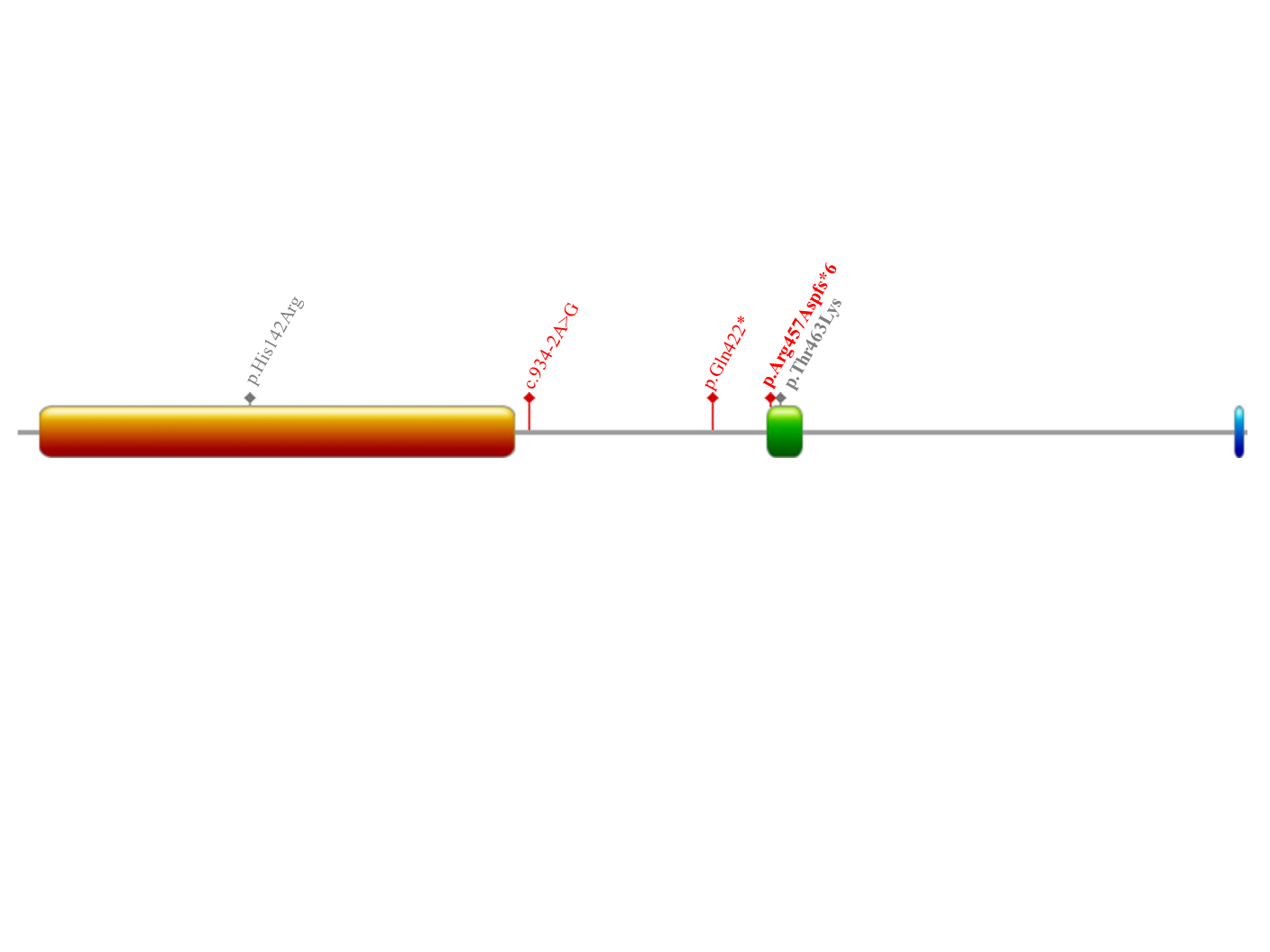

Supplement: Supplementary file 2 — Figure S2. The protein is composed of a protein kinase domain (orange), a leucine zipper region (green) and the NEMO binding-region (blue). Missense (gray) and loss of function (red) pathogenic variants found in the CHUK gene. Reported in this study (Bold). (TIFF 128 kb) [file 12881_2018_556_MOESM2_ESM.tif]

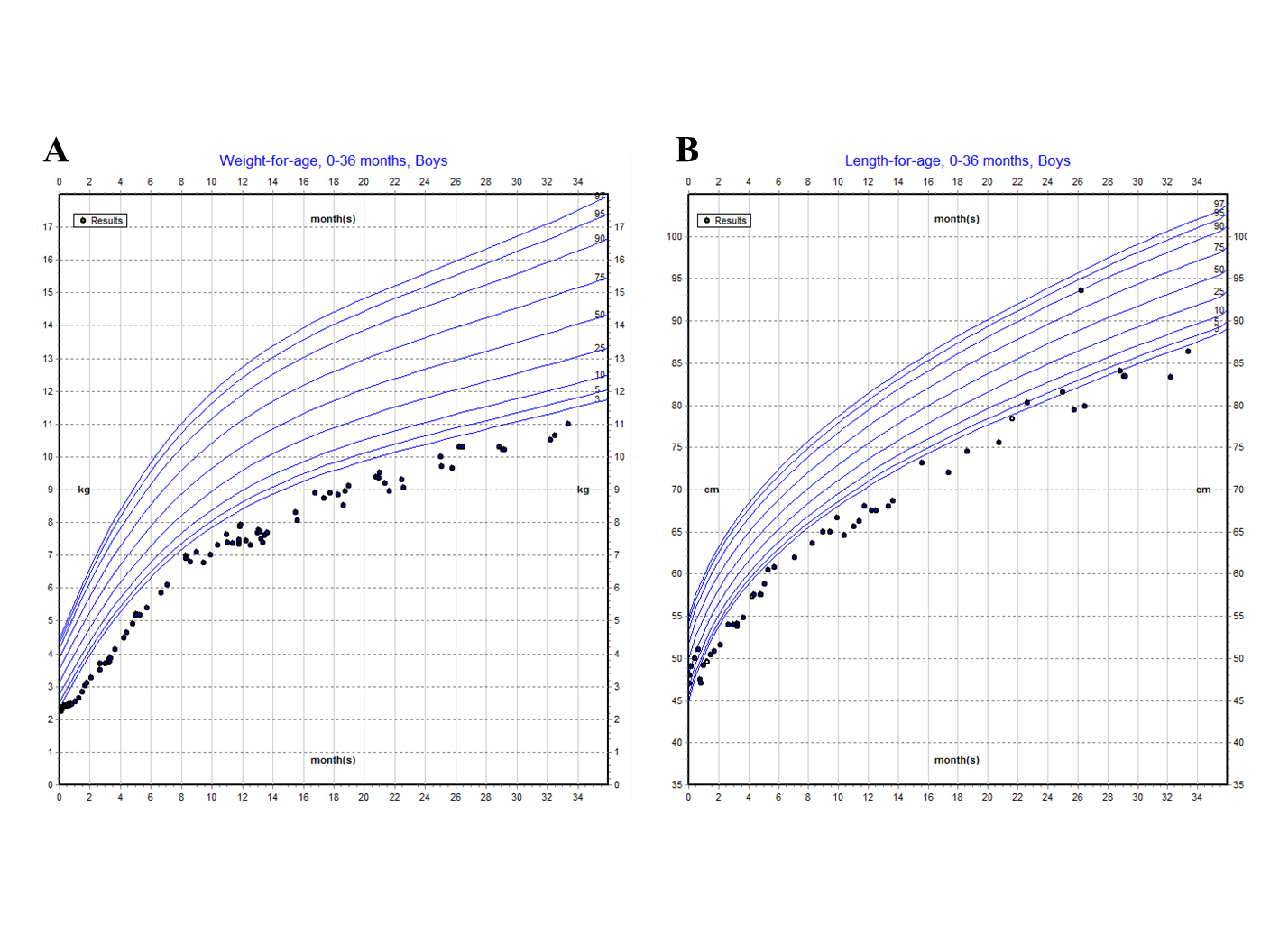

Supplement: Supplementary file 3 — Figure S3. Growth chart. A) Weight for age (kg). B) Length for age (cm). (TIFF 602 kb) [file 12881_2018_556_MOESM3_ESM.tif]
